# Impact of a Meds-to-Beds PCSK9i Initiation Program on LDL-C in Patients Undergoing ASCVD Revascularization

**DOI:** 10.1016/j.jacadv.2025.102075

**Published:** 2025-08-19

**Authors:** Daniel Lorenzatti, Garred S. Greenberg, Annalisa Filtz, Andrea Scotti, Vita N. Jaspan, Christine M. Park, Bethany Kalich, Laney K. Jones, Niranjan Kathe, Katherine E. Di Palo, Constance Browne, Evan Lipsitz, Stephen J. Forest, Azeem Latib, Mario J. Garcia, Carlos J. Rodriguez, Michael D. Shapiro, Martha Gulati, Leandro Slipczuk

**Affiliations:** a Division of Cardiology, Montefiore Health System/Albert Einstein College of Medicine, Bronx, New York, USA; b Amgen Inc, Thousand Oaks, California, USA; c Division of Hospital Medicine, Montefiore Health System/Albert Einstein College of Medicine, Bronx, New York, USA; d Vascular Surgery Division, Montefiore Medical Center/Albert Einstein College of Medicine, Bronx, New York, USA; e Department of Cardiothoracic & Vascular Surgery, Montefiore Medical Center/Albert Einstein College of Medicine, Bronx, New York, USA; f Cardiothoracic Surgery Division, Montefiore Medical Center/Albert Einstein College of Medicine, Bronx, New York, USA; g Section of Cardiovascular Medicine, Center for Prevention of Cardiovascular Disease, Wake Forest University School of Medicine, Winston-Salem, North Carolina, USA; h The Barbra Streisand Women’s Heart Center, Smidt Heart Institute, Cedars-Sinai Medical Center, Los Angeles, California, USA; i The Baim Institute for Clinical Research, Boston, Massachusetts, USA

**Keywords:** ASCVD, LDL-C, PCSK9 inhibitors, non–HDL-C, meds-to-beds

## Abstract

**Background:**

Despite proven benefits of low-density lipoprotein cholesterol (LDL-C) lowering in secondary prevention of atherosclerotic cardiovascular disease, goal achievement remains suboptimal.

**Objectives:**

The authors tested whether early use of guideline-recommended proprotein convertase subtilisin/kexin type 9 inhibitor (PCSK9i) monoclonal antibodies (mAbs) through a meds-to-beds (M2B) program improved LDL-C goal attainment postrevascularization.

**Methods:**

Using a dedicated M2B program, we prospectively included patients undergoing coronary or peripheral artery revascularization, on maximally tolerated statin therapy, and with LDL-C ≥70 mg/dL. Patients received support to start PCSK9i mAbs and were followed for at least 6 months. LDL-C goal attainment rates were compared with standard of care using a direct-matched retrospective cohort. Statistical comparisons were made with chi-squared, Wilcoxon rank sum, and *t*-tests, as appropriate.

**Results:**

The 72 patients in the prospective PCSK9i mAb cohort (median age 66 years, 38% women, 57% Hispanic) were matched to 136 historical controls. Baseline median LDL-C was 96 mg/dL (IQR: 80, 122) in the PCSK9i mAb group and 109 mg/dL (IQR: 87, 137) in the control group (*P* < 0.05). At 6 months, LDL-C goal achievement was greater in the PCSK9i mAb group (92% achieved LDL-C <70 mg/dL and 79% achieved LDL-C <55 mg/dL) than in the control group (40% and 25%, respectively) (*P* < 0.001 for both). A larger median percent reduction in LDL-C was also observed in the PCSK9i mAb group than in the historical control group (66% vs 25%; *P* < 0.001).

**Conclusions:**

Early initiation of guideline-recommended PCSK9i mAbs through a dedicated M2B program was associated with enhanced attainment of LDL-C goals in patients with established atherosclerotic cardiovascular disease undergoing revascularization.

Atherosclerotic cardiovascular disease (ASCVD) remains the leading global cause of all-cause and cardiovascular (CV) mortality, responsible for approximately one-third of all deaths.^1^ Elevated low-density lipoprotein cholesterol (LDL-C) remains a major modifiable risk factor for ASCVD and is directly linked to worse patient outcomes. In individuals with established ASCVD, elevated LDL-C significantly increases the risk for recurrent major adverse cardiovascular events.^2^^,^^3^ For secondary prevention of ASCVD, current guidelines recommend an LDL-C level <70 mg/dL in patients with established ASCVD and a target of <55 mg/dL for those with ASCVD who are considered very high risk.^4^, ^5^, ^6^ Extensive randomized clinical trials have demonstrated the clinical benefits of LDL-C lowering using lipid-lowering therapies (LLTs).^7^, ^8^, ^9^, ^10^

Statin therapy remains the cornerstone of LDL-C lowering to reduce the risk of CV events in patients with established ASCVD; however, recent data show that approximately 70% to 85% of adults with ASCVD have LDL-C levels ≥70 mg/dL.^11^^,^^12^ Among patients receiving LLT, LDL-C levels of <70 mg/dL are achieved in only 20% to 35% of individuals, depending on their baseline LDL-C levels.^13^ Furthermore, among individuals with ASCVD and LDL-C levels ≥100 mg/dL, only 22% undergo LLT intensification after 2 years of follow-up.^13^ Critical barriers to achieving optimal and sustained LDL-C reductions include clinical inertia, medication nonadherence, and limited access to therapy.^14^ Meds-to-beds (M2B) programs have been shown to improve access and patient adherence to therapies, leading to improved outcomes.^15^ Usually, these programs have 3 core components: delivering medications directly to the patient’s bedside before discharge, providing comprehensive patient education on the prescribed medications, and offering medications at no cost to uninsured or underinsured patients.

Proprotein convertase subtilisin/kexin type 9 inhibitor (PCSK9i) monoclonal antibodies (mAbs) were first approved by the Food and Drug Administration for LLT intensification in 2015. These agents have demonstrated rapid, profound, and consistent reductions in LDL-C levels across the whole spectrum of coronary disease, including primary^16^^,^^17^ and secondary prevention,^7^^,^^18^ when added to statins alone or in combination with ezetimibe. Despite long-standing regulatory approval, inclusion in guideline recommendations, recent significant cost reduction, and relatively broad insurance coverage, their real-world adoption has been limited.^19^ A recent analysis of a large electronic medical record database revealed 99.5% of patients with LDL-C levels ≥70 mg/dL who are eligible for PCSK9i mAbs are not receiving a prescription for these therapies.^20^ Furthermore, denied or abandoned PCSK9i mAb claims are linked to higher CV event risk, highlighting the negative impact of limited access on clinical outcomes.^21^

To date, there are limited data on the use of PCSK9i mAbs through an M2B approach and the impact of this strategy on LDL-C levels at follow-up.^22^ In this study, we aimed to assess the effect of early initiation of PCSK9i mAbs through an M2B program compared with standard of care on LDL-C goals in the first 6 months following revascularization for ASCVD.

## Methods

### Study design

The ELL-ASCVD (Early Lipid Lowering after ASCVD) study was a prospective, open-label, interventional, single-center study with a direct-matched retrospective historical control group. The Office of Human Research Affairs at Albert Einstein College of Medicine Institutional Review Board approved the study and waived the requirement to obtain informed consent for the retrospective cohort. All patients included in the prospective group gave written or verbal consent to participate. The study protocol was developed in accordance with the SPIRIT (Standard Protocol Items: Recommendations for Interventional Trials) guidelines. The completed SPIRIT checklist is available in the Supplemental Table 1.

### M2B program

The M2B program was established in 2019 with a collaboration between the Department of Cardiology and the hospital pharmacy, aimed at improving prescription of and adherence to dual antiplatelet therapy in patients postrevascularization. In June 2023, the program was expanded to optimize the use of guideline-recommended PCSK9i mAbs in patients with suboptimal LDL-C in this selected population. Since then, the M2B PCSK9i mAbs program has involved the following: LDL-C testing to identify patients not at LDL-C goal, prescribing PCSK9i mAbs, facilitating prior authorizations for insurance, providing financial assistance when applicable, arranging medication delivery to the bedside or patient’s home, and educating patients on proper medication use. While the program assisted with completing insurance paperwork and coordinating prior authorizations, it did not provide free medications or direct financial assistance, as it was designed to reflect a real-world model potentially replicable across other health systems.

### Prospective cohort

The prospective arm included patients ≥18 years of age admitted to Montefiore Health System–Albert Einstein College of Medicine (Bronx, New York), a quaternary, large health system, from June 2023 to February 2024 for an ASCVD revascularization procedure involving the coronary arteries, lower extremities, or carotid arteries. All surgical (bypass, endarterectomy) or percutaneous (angioplasty) as well as planned or emergency interventions were considered.

To be eligible, patients had to have: 1) a serum LDL-C ≥70 mg/dL, measured either within 1 month prior to the index procedure or prior to discharge during the index procedure; 2) been on maximally tolerated statin therapy for at least 4 weeks, as determined by the investigator upon interview and chart review. Statin-intolerant patients were eligible if they had side effects related to at least 2 different statins.

Patients were not eligible if they met any of the following criteria:

1. Any uncontrolled or serious disease or any medical or surgical condition that may interfere with the administration of PCSK9i mAbs.
2. Severe concomitant non-CV disease that carries the risk of reducing life expectancy to <1 year.
3. Receiving PCSK9i mAb or inclisiran prior to index date.
4. Pregnant women.
5. Known history of alcohol and/or drug abuse within the last 1 year (as per investigator discretion).
6. History of hypersensitivity to any of the study treatments (PCSK9i mAbs) or its excipients.
7. Planned use of other investigational products or devices during the course of the study.
8. Active liver disease, defined as any known current infectious, neoplastic, or metabolic pathology of the liver, or alanine aminotransferase elevation >3× upper limit of normal (ULN), aspartate aminotransferase elevation >3× ULN, or total bilirubin elevation >2× ULN at screening.
9. Death during the index procedure/hospitalization.
10. No medical records before the index procedure.
11. Missing information regarding key variables, namely age, sex, type of ASCVD revascularization.

Patients meeting the inclusion criteria were approached for consent during admission and were prescribed PCSK9i mAbs through the M2B program. Depending on the time required to obtain prior authorization from the insurance company and the length of hospital stay, medications were either delivered to the bed or to the patient’s home/pharmacy, according to their preference. Patients received education on proper medication use before discharge, which was reinforced within 30 days postdischarge through either a follow-up telephone call or an in-person visit to the cardiology office. All patients were followed for a minimum of 6 months after the index procedure. LDL-C was measured as part of routine clinical care during follow-up. For analysis, the first available measurement obtained at least 30 days after therapy initiation and within 1 year of the index procedure was used. Patients without an LDL-C measurement in this time frame were considered lost to follow-up.

### Retrospective control cohort

**Figure 1:**
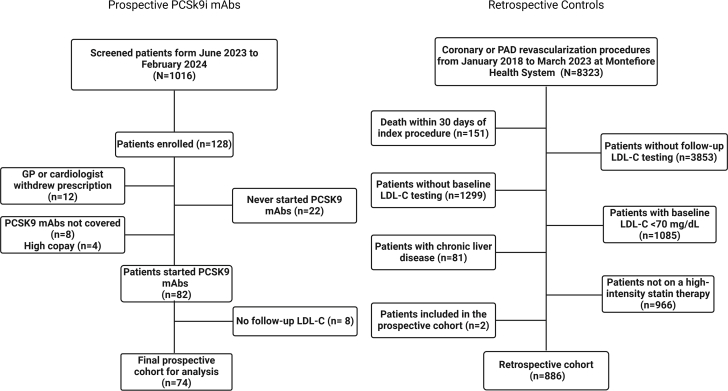
Flow Diagrams of the Study Patients GP = general practitioner; LDL-C = low-density lipoprotein cholesterol; mAbs = monoclonal antibodies; PAD = peripheral artery disease; PCSK9i = proprotein convertase subtilisin/kexin type 9 inhibitor.

Controls were selected using direct matching methods from a retrospective cohort of patients ≥18 years of age who had undergone revascularization for either coronary artery disease or peripheral artery disease, from January 2018 to March 2023 at Montefiore Health System–Albert Einstein College of Medicine (N = 8,323). This retrospective cohort was restricted to 4,319 patients who had baseline and follow-up LDL-C testing within the health system after their index procedure. Patients were excluded if they did not have a documented baseline LDL-C measurement, defined as an LDL-C measured 30 days before to 1 week after the index procedure, and follow-up LDL-C, defined as an LDL-C measured at least 30 days after the index measurement. Patients also had to be on high-intensity statin therapy, defined as atorvastatin 40 mg or 80 mg, or rosuvastatin 20 mg or 40 mg once daily, for at least 30 days before the baseline LDL-C measurement (Figure 1).

### Matching algorithms

The matching process was prespecified in the study protocol and implemented using the MatchIt package in R v4.0.3 (R Foundation for Statistical Computing). Patients in the PCSK9i mAb cohort were matched to retrospective controls using a 1:2 ratio. The primary matching algorithm used an exact match on procedure type and sex, and greedy nearest-neighbor matching on age, without replacement. Two cases in the PCSK9i mAb cohort did not have a match, and 8 had only one match rather than 2. To assess robustness, we conducted a secondary expanded matching using a 1:2 ratio with exact matching on procedure type and greedy nearest-neighbor matching on the following covariates: age, age squared, sex, race/ethnicity, insurance type, hypertension (HTN), diabetes mellitus, obesity, coronary artery disease, myocardial infarction (MI), congestive heart failure, atrial fibrillation, peripheral vascular disease, chronic kidney disease, lung disease, substance use, baseline LDL-C, and baseline LDL-C squared (Supplemental Table 2). Covariate balance before and after matching was assessed using absolute standardized mean differences. Love plots illustrating this balance for both the primary and alternative matching models are provided in Supplemental Figure 1.

### Data abstraction

Data were abstracted using a contemporary data platform integrated with our institution’s electronic health record, built on an open-source technology developed by Observational Health Data Sciences and Informatics, an international coalition of health data informaticians, scientists, and researchers.^23^ Using the Observational Medical Outcomes Partnership Common Data Model, Observational Health Data Sciences and Informatics’s open community data standard for the structure and content of observational data, the data platform can adopt common terminologies and coding schemes across disparate databases and integrate demographic, clinical, laboratory, and prescription data.^24^^,^^25^ This system has previously been known to capture most events, with readmissions outside Montefiore occurring rarely (1.9%).^26^

### Outcomes

The study's primary outcomes were the percentage of patients achieving LDL-C <70 mg/dL and percentage achieving LDL-C <55 mg/dL. The secondary outcomes included: 1) median change in LDL-C levels from baseline to follow-up; and 2) median percentage of patients who reached non–high-density lipoprotein cholesterol (HDL-C) <100 mg/dL and <85 mg/dL at follow-up.

### Statistical analysis

Continuous data are shown as median (IQRs) and categorical data are shown as absolute numbers (percentages). Comparisons across treatment groups were analyzed using the *t*-test, chi-squared, Wilcoxon rank sum, and Fisher exact tests, as appropriate. For each outcome, multivariable logistic regression was conducted using the direct matched cohort to assess the impact of early PCSK9i mAb initiation on the odds of outcome attainment. Clinical variables that were significantly different between the PCSK9i mAb cohort and the retrospective control group were included as controls in the multivariate models. Given the high frequency of goal attainment in the intervention group, a Poisson regression model with robust standard errors was additionally performed to estimate risk ratios, adjusting for the same covariates included in the primary logistic regression. As a sensitivity analysis, we constructed a fully adjusted logistic regression model that included all available clinical covariates, excluding baseline total cholesterol and non–HDL cholesterol due to collinearity with LDL-C. This model was used to assess the robustness of the primary findings. A linear mixed-effects model adjusting for prior MI, HTN, baseline LDL-C, baseline triglycerides, ezetimibe use, and high-intensity statin use, as well as clustering within matched data, was used to estimate adjusted mean differences in LDL-C. A *P* value of <0.05 was considered statistically significant. Statistical analyses were performed using R version 4.0.3 (R Foundation for Statistical Computing).

## Results

### Study population

Of the 128 patients initially included, only 82 were able to start PCSK9i mAbs and 74 completed the follow-up in the prospective group (Figure 1). After direct matching the PCSK9i mAb cohort with the historical control group (1:2 ratio), the final analyzed population comprised 72 patients from the PCSK9i mAb group and 136 standard-of-care historical controls. Only 2 patients from the PCSK9i mAb cohort could not be matched, and 8 individuals from the peripheral artery disease revascularization subgroup had only 1 available match. Baseline clinical characteristics are summarized in Table 1. As expected, there were no major differences between the 2 groups for age, sex, and procedure type. The median age was 66 years (IQR: 60, 71) and the majority of patients were male (63%). Percutaneous coronary intervention was the most common procedure (72%). There were no major differences in race/ethnicity or insurance type between the PCSK9i mAb cohort and historical control group. ASCVD risk factors and comorbidities were also similarly distributed, except for HTN and history of MI, which were significantly more prevalent in the historical control group (99% vs 93%; *P* = 0.02 and 79% vs 53%; *P* < 0.001, respectively) (Table 1).

**Table 1:** Characteristics of the Study Population After Direct Matching

Variable	PCSK9i mAbs Group (n = 72)	Historical Control Group (n = 136)	Overall (N = 208)	*P* Value^a^
Age (y)	66 (59, 71)	66 (61, 72)	66 (60, 71)	0.70
Sex				>0.9
Female	27 (38%)	50 (37%)	77 (37%)	
Male	45 (63%)	86 (63%)	131 (63%)	
Procedure				0.50
PCI	50 (69%)	100 (74%)	150 (72%)	
CABG	13 (18%)	26 (19%)	39 (19%)	
PAD	9 (13%)	10 (7.4%)	19 (9.1%)	
Race/ethnicity				0.091
Hispanic	41 (57%)	61 (45%)	102 (49%)	
Non-Hispanic Black	11 (15%)	39 (29%)	50 (24%)	
Non-Hispanic White	10 (14%)	12 (8.8%)	22 (11%)	
Other/unknown	10 (14%)	24 (18%)	34 (16%)	
Insurance type				0.081
Medicare	23 (32%)	35 (26%)	58 (28%)	
Medicaid	26 (36%)	53 (39%)	79 (38%)	
Private	15 (21%)	43 (32%)	58 (28%)	
Unknown	8 (11%)	5 (3.7%)	13 (6.3%)	
Hypertension	67 (93%)	135 (99%)	202 (97%)	**0.020**
Diabetes mellitus	51 (71%)	99 (73%)	150 (72%)	0.80
Obesity	33 (46%)	66 (49%)	99 (48%)	0.70
CAD	70 (97%)	136 (100%)	206 (99%)	0.12
Myocardial infarction	38 (53%)	107 (79%)	145 (70%)	**<0.001**
CHF	33 (46%)	70 (51%)	103 (50%)	0.40
Atrial fibrillation	12 (17%)	27 (20%)	39 (19%)	0.60
Peripheral vascular disease	27 (38%)	59 (43%)	86 (41%)	0.40
Chronic kidney disease	30 (42%)	58 (43%)	88 (42%)	0.90
Chronic lung disease	31 (43%)	61 (45%)	92 (44%)	0.80
Substance use	24 (33%)	57 (42%)	81 (39%)	0.20
Baseline statin	65 (90%)	136 (100%)	201 (97%)	**<0.001**
Baseline high-intensity statin	64 (89%)	136 (100%)	200 (96%)	**<0.001**
Baseline ezetimibe	20 (28%)	13 (9.6%)	33 (16%)	**<0.001**
Follow-up statin	62 (86%)	131 (96%)	193 (93%)	**0.007**
Follow-up high-intensity statin	61 (85%)	125 (92%)	186 (89%)	0.11
Follow-up ezetimibe	21 (29%)	25 (18%)	46 (22%)	0.075
Follow-up PCSK9i mAbs	72 (100%)	12 (8.8%)	84 (40%)	**<0.001**

Values are median (Q1, Q3) or n (%). Values in **bold** indicate statistical significance (*P* < 0.05).

CABG = coronary artery bypass graft; CAD = coronary artery disease; CHF = congestive heart failure; mAbs = monoclonal antibodies; PAD = peripheral artery disease; PCI = percutaneous coronary intervention; PCSK9i = proprotein convertase subtilisin/kexin type 9 inhibitor.

a Wilcoxon rank sum test, Pearson's chi-squared test, or Fisher exact test.

### Medication use

LLTs at baseline and follow-up are reported in Table 1. There was a significant difference in statin use at baseline (*P* < 0.001), with a total of 65 patients (90%) in the PCSK9i mAb cohort on statin therapy and 64 (89%) on a high-intensity statin, whereas all 136 patients (100%) were on a high-intensity statin in the historical control group. There was also a significant difference in ezetimibe use at baseline (*P* < 0.001), with 20 patients (28%) in the PCSK9i mAb cohort receiving ezetimibe at baseline compared with 13 patients (9.6%) in the historical control group. None of the included patients were using PCSK9i mAbs at baseline in any group (Table 1).

### Lipid panel testing

All baseline and follow-up lipid measurements are summarized in Table 2. The median time to follow-up LDL-C testing was significantly shorter in the PCSK9i mAb cohort vs historical control group (2 [IQR: 2, 4] vs 13 [IQR: 4, 30] months, *P* < 0.001) (Table 2). Additionally, LDL-C was measured in 89% of patients in the PCSK9i mAb cohort within 6 months, compared to 37% in the control group (*P* < 0.001), with similar observations at 90 days (60% vs 23%) and 1 year (99% vs 49%; both *P* < 0.001).

**Table 2:** Baseline and Follow-Up Lipid Measurements

Lipid Measurement	PCSK9i mAbs Group (n = 72)	Historical Control Group (n = 136)	Overall (N = 208)	*P* Value^a^
Baseline LDL-C (mg/dL)	96 (80, 123)	109 (87, 137)	107 (83, 132)	**0.049**
Baseline non–HDL-C (mg/dL)	117 (99, 153)	137 (110, 169)	133 (107, 166)	**0.022**
Baseline HDL-C (mg/dL)	39 (34, 47)	42 (34, 49)	42 (34, 48)	0.30
Baseline total cholesterol (mg/dL)	160 (140, 198)	177 (155, 212)	171 (147, 208)	**0.005**
Baseline triglycerides (mg/dL)	107 (80, 146)	123 (90, 176)	115 (85, 168)	**0.041**
Follow-up LDL-C (mg/dL)	33 (23, 50)	77 (55, 110)	60 (40, 100)	**<0.001**
Follow-up non–HDL-C (mg/dL)	62 (45, 92)	105 (81, 147)	92 (63, 132)	**<0.001**
Follow-up HDL-C (mg/dL)	41 (36, 51)	40 (34, 49)	40 (35, 49)	0.40
Follow-up total cholesterol (mg/dL)	105 (87, 129)	144 (120, 199)	132 (104, 181)	**<0.001**
Follow-up triglycerides (mg/dL)	111 (87, 154)	111 (77, 171)	111 (82, 167)	0.80
Follow-up LDL-C within 90 d (n)	43 (60%)	31 (23%)	74 (36%)	**<0.001**
Follow-up LDL-C within 6 mo (n)	64 (89%)	50 (37%)	114 (55%)	**<0.001**
Follow-up LDL-C within 1 y (n)	71 (99%)	67 (49%)	138 (66%)	**<0.001**
Months to follow-up LDL-C	2 (2, 4)	13 (4, 30)	5 (2, 23)	**<0.001**

Values are median (Q1, Q3) or n (%). Values in **bold** indicate statistical significance (*P* < 0.05).

HDL-C = high-density lipoprotein cholesterol; LDL-C = low-density lipoprotein cholesterol; other abbreviations as in Table 1.

a Wilcoxon rank sum test or Pearson's Chi-squared test.

The historical control group had significantly higher baseline median LDL-C (109 [IQR: 87, 137] vs 96 [IQR: 80, 123] mg/dL, *P* = 0.049), total cholesterol (177 [IQR: 155, 212] vs 160 [IQR: 140, 198] mg/dL, *P* = 0.005), and triglycerides (123 [IQR: 90, 176] vs 107 [IQR: 80, 146] mg/dL, *P* = 0.041) compared with the PCSK9i mAb cohort, while baseline median HDL-C showed no significant difference.

### Outcomes

**Figure 2:**
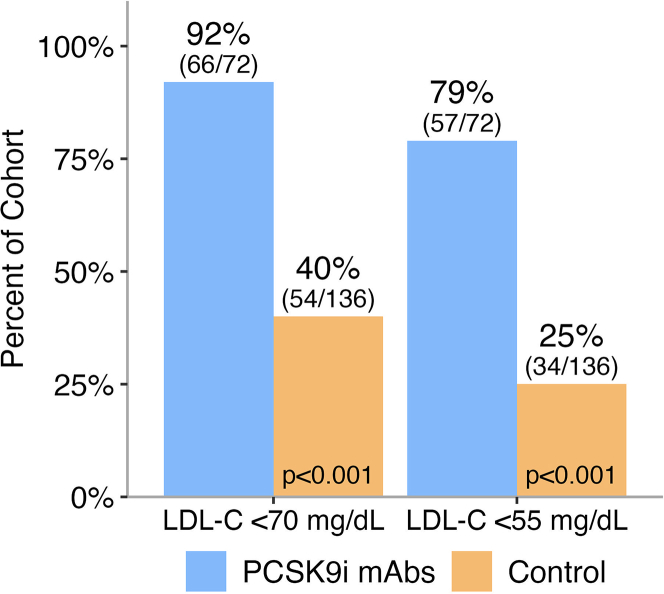
Percentage of Patients With LDL-C Goal Attainment at ≥6 Month Follow-Up Abbreviations as in Figure 1.

**Figure 3:**
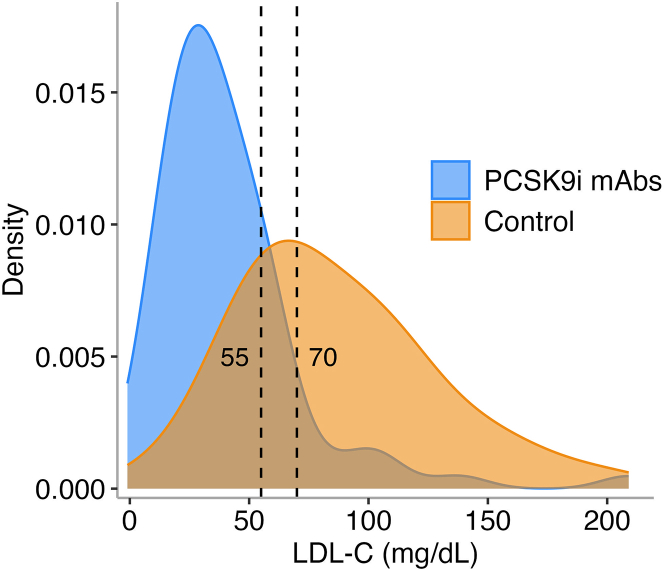
Density Plot of LDL-C Values at ≥6 Month Follow-Up Abbreviations as in Figure 1.

**Figure 4:**
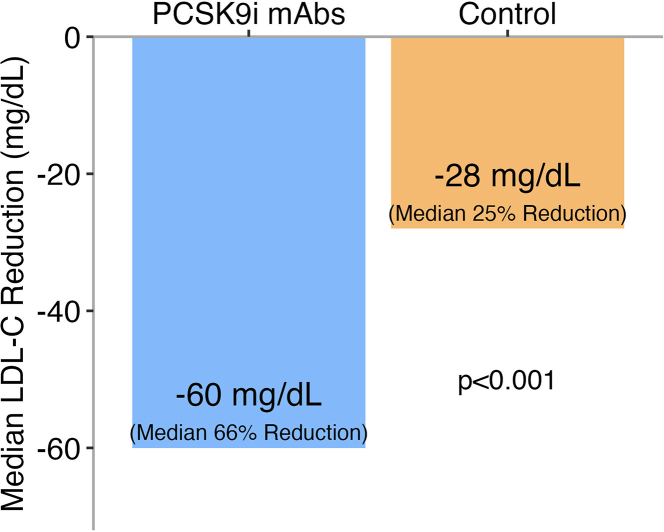
Median LDL-C Reduction From Baseline to ≥6 Month Follow-Up Abbreviations as in Figure 1.

**Figure 5:**
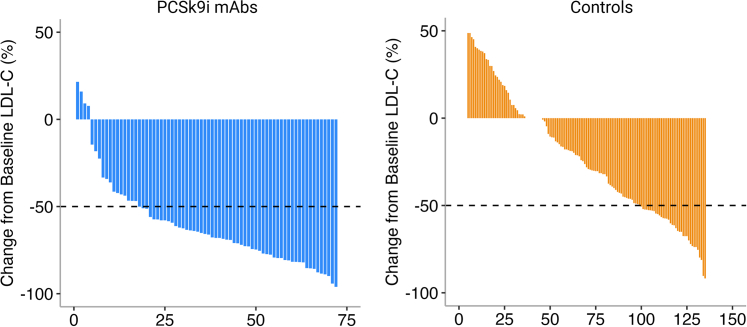
Waterfall Plots Showing Distribution of Percentage Change in LDL-C at ≥6 Month Follow-Up Abbreviations as in Figure 1.

Primary and secondary outcomes are reported in Table 3. The goal of LDL-C <70 mg/dL was achieved in 66 patients (92%) in the PCSK9i mAb cohort compared with 54 patients (40%) in the historical control group (*P* < 0.001). An LDL-C <55 mg/dL was achieved in 57 patients (79%) in the PCSK9i mAb cohort compared with 34 patients (25%) in the historical control group (*P* < 0.001) (Figure 2). At follow-up, the median LDL-C (IQR) was 33 (23, 50) mg/dL in the PCSK9i mAb cohort, compared with 77 (55, 110) mg/dL in the historical control group (Table 2, Figure 3). This change represented a median reduction (IQR) of 66% (50, 78) for the PCSK9i mAb cohort, compared with 25% (−2, 52) in the historical control group (*P* < 0.001) (Figure 4, Figure 5, Table 3). In the adjusted mixed-effects model, the intervention group had a 47.2 mg/dL greater reduction in LDL-C compared to usual care (SE: 6.2 mg/dL; *P* < 0.001). Among the patients initially enrolled in the study, only 6.3% (n = 8) were denied coverage by insurance, and 17.2% (n = 22) had abandoned prescriptions in the PCSK9i mAb cohort (Figure 1).

**Table 3:** Outcome Attainment by Group

Endpoint	PCSK9i mAbs Group (n = 72)	Historical Control Group (n = 136)	Overall (N = 208)	*P* Value^a^
Follow-up LDL-C <70 mg/dL	66 (92%)	54 (40%)	120 (58%)	**<0.001**
Follow-up LDL-C <55 mg/dL	57 (79%)	34 (25%)	91 (44%)	**<0.001**
Median change in LDL-C (IQR)	−60 (−91, −44)	−28 (−65, 2)	−44 (−73, −9)	**<0.001**
Reduction in LDL-C (%)	66 (50, 78)	25 (−2, 52)	45 (7, 65)	**<0.001**
Follow-up non–HDL-C <100 mg/dL	55 (79%)	61 (45%)	116 (56%)	**<0.001**
Follow-up non–HDL-C <85 mg/dL	51 (73%)	44 (32%)	95 (46%)	**<0.001**

Values are n (%) or median (Q1, Q3). Values in **bold** indicate statistical significance (*P* < 0.05).

Abbreviations as in Table 1, Table 2.

a Pearson's chi-squared test or Wilcoxon rank sum test.

**Figure 6:**
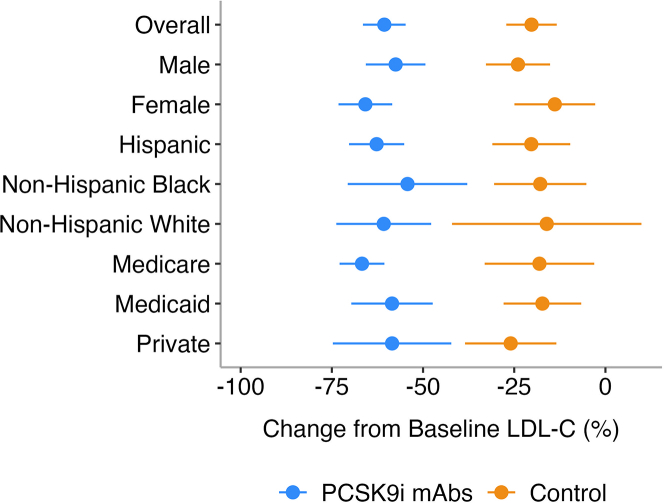
Mean Percentage Change in LDL-C From Baseline to ≥6 Month Follow-Up Across Different Subgroups LDL-C reduction was consistently greater with PCSK9i mAbs across all subgroups (*P* < 0.05). No significant differences were observed between subgroups within either group. Abbreviations as in Figure 1.

When adjusted for all the characteristics that were significantly different at baseline between the groups, the PCSK9i mAb cohort was associated with significantly higher odds of attaining guideline-directed goals for both LDL-C and non-HDL-C (Table 4). These findings remained consistent in an alternative matching approach that incorporated an expanded set of clinical variables, including baseline LDL-C, with persistently greater reductions in lipid levels and higher goal attainment rates in the intervention group (Supplemental Table 2). Results were further reinforced in an adjusted sensitivity model that accounted for all available covariates, showing a comparable treatment effect and model fit relative to the primary analysis (Supplemental Table 3). To improve interpretability in the context of a common outcome, risk ratios were estimated using a Poisson regression model with robust standard errors yielding similar results and supporting the robustness of the treatment effect (Supplemental Table 4). Lastly, in prespecified subgroup analyses by sex, race/ethnicity, and insurance type, the LDL-C reduction was consistent across all evaluated subgroups (Figure 6).

**Table 4:** Multivariate Analysis of the Intervention Impact on Goal Attainment

	aOR (95% CI)
LDL-C <70 mg/dL	29.7 (9.8-122.9)
LDL-C <55 mg/dL	15.5 (6.8-39.4)
Non–HDL-C <100 mg/dL	7.0 (3.1-17.3)
Non–HDL-C <85 mg/dL	8.8 (4.0-21.2)

Abbreviations as in Table 2.

a Adjusted for variables that were statistically different between groups after matching: HTN, MI, baseline high-intensity statin, baseline ezetimibe, baseline LDL-C, baseline TG. Baseline non−HDL-C and baseline cholesterol were both removed from the model due to 93% collinearity with LDL-C.

## Discussion

This prospective study found that an M2B program administering PCSK9i mAbs and targeting recently revascularized patients with suboptimal LDL-C despite maximally tolerated statins significantly reduced LDL-C levels and improved goal attainment (<70 mg/dL and <55 mg/dL) at 6 months compared to standard care (Central Illustration).

**Central Illustration:**
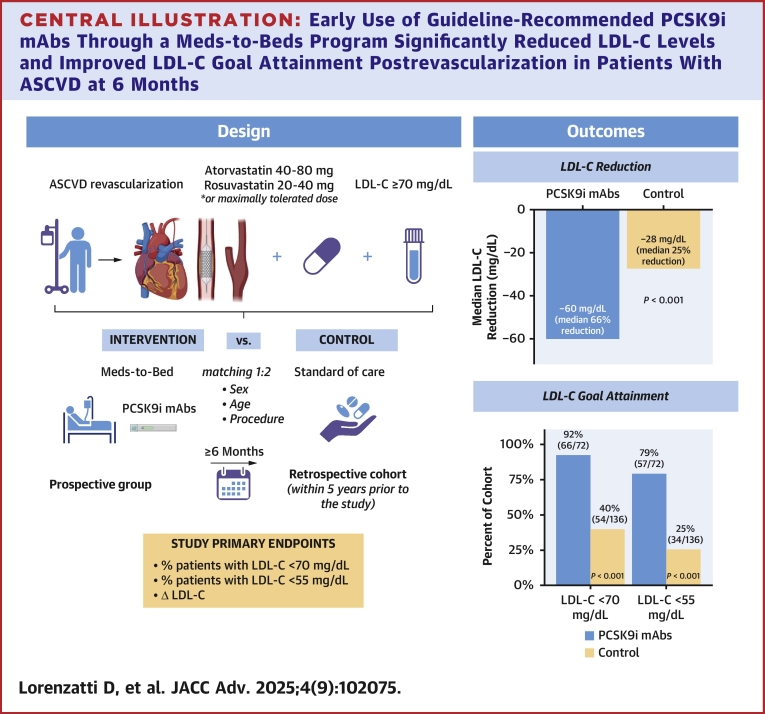
Early Use of Guideline-Recommended PCSK9i mAbs Through a Meds-to-Beds Program Significantly Reduced LDL-C Levels and Improved LDL-C Goal Attainment Postrevascularization in Patients With ASCVD at 6 Months ASCVD = atherosclerotic cardiovascular disease; other abbreviations as in Figure 1.

Despite the large CV outcomes trials^7^^,^^18^^,^^27^ and guideline recommendations,^6^ the majority of patients with established ASCVD and an LDL-C ≥70 mg/dL do not receive appropriate LLT intensification in real-world practice.^13^^,^^28^ Commonly cited barriers such as clinical inertia, medication adherence issues, and disparities in access to therapy may partly explain this phenomenon.^14^ While high costs were historically seen as a barrier to PCSK9i mAb use, a 60% price reduction has not resulted in the expected increase in uptake.^13^ Consequently, the implementation of hospital programs to overcome some of the noncost barriers is crucial to minimize and/or prevent adverse clinical outcomes in patients with established ASCVD.^14^ It has also been demonstrated that multiple bundled interventions are more effective than individual interventions alone;^29^ however, the impact of M2B programs in terms of clinical outcomes has been controversial.^30^ A systematic review of 17 studies evaluating the impact of multilevel pharmacist care in the setting of postacute coronary syndrome did not demonstrate any significant improvement in medication adherence or reductions in readmissions, emergency visits, and mortality.^31^ In contrast, our study demonstrated the implementation of a comprehensive M2B program prescribing PCSK9i mAbs for recently revascularized patients with suboptimal LDL-C despite maximally tolerated statin led to significant improvement in key CV indicators such as LDL-C reduction and goal attainment.

Large randomized clinical trials^7^^,^^18^ and real-world studies^32^^,^^33^ have demonstrated that PCSK9i mAbs effectively lower LDL-C and reduce CV risk, but access remains limited for many patients. Furthermore, rejected and abandoned prescriptions have been shown to negatively impact clinical outcomes.^21^ In the PCSK9i mAb cohort, we observed a very low rate of insurance denials (6.3%) and a rate of abandoned prescriptions (17.2%) similar to what has been previously reported.^21^ Lack of LDL-C testing in patients requiring secondary prevention may also lead to missed opportunities for statin intensification and nonstatin therapy use.^34^ We have shown previously that female sex, non-Hispanic Black race/ethnicity, and delayed follow-up testing are independently associated with lower likelihood of achieving optimal LDL-C levels.^35^ In this study, we observed different patterns of LDL-C testing between the PCSK9i mAb cohort compared with the historical standard-of-care group. Most (89%) of the follow-up LDL-C measurements in the PCSK9i mAb cohort were conducted within 6 months from the index procedure, compared with only 37% in the historical control group. Identifying and addressing specific barriers to PCSK9i mAb access can reduce the clinical and economic burden for patients who would benefit from these treatments.^36^

Even though the safety and effectiveness of PCSK9i mAbs for LDL-C reduction have been demonstrated in randomized clinical trials^37^ and retrospective real-world cohorts,^32^^,^^33^ the novelty of our study lies in the prospective nature of the intervention in a real-world setting and the use of a dedicated M2B program to identify patients not at LDL-C goal and enhance medication access. In a simulation study using the DAVINCI (EU-Wide Cross-Sectional Observational Lipid-Modifying Therapy Use in Secondary and Primary Care) study, Brandts et al reported that the potential use of a combined therapy including an optimized statin, ezetimibe, and a PCSK9i would result in approximately 90% of patients achieving their risk-based LDL-C goals.^38^ We found a similar rate of improvement in the proportion of patients achieving both LDL-C <70 mg/dL and <55 mg/dL goals, with the majority of patients being on a statin and PCSK9i mAb and only 30% with concomitant ezetimibe use.

In addition to significantly improved LDL-C goal achievement in the PCSK9i mAb cohort, there was also a notable reduction in non–HDL-C levels, including all atherogenic (apolipoprotein B-containing) lipoproteins, which are strongly linked with increased CV risk.^39^ Accordingly, non–HDL-C goals are also recommended as targets by clinical practice guidelines.^4^^,^^40^ Furthermore, the SWEDEHEART (Swedish Web-System for Enhancement and Development of Evidence-Based Care in Heart Disease Evaluated According to Recommended Therapies) MI registry, which included over 56,000 patients, showed that despite optimized statin therapy, about 80% of post-MI patients do not reach LDL-C targets and that greater non–HDL-C reductions are achieved with the use of combination LLT.^41^

Although the M2B program facilitated access to PCSK9i mAbs therapy by streamlining prior authorizations and ensuring bedside or home delivery, it did not provide medications free of charge or direct financial assistance, as it was designed to reflect a real-world model replicable across institutions without dedicated funding. In 12 cases, the prescription was withdrawn by the primary care provider, reflecting either disagreement with the guideline-recommended therapy or a preference to pursue alternative approaches. Despite the M2B program’s assistance in facilitating insurance approval and navigating some access barriers, 8 patients were not covered by insurance and 4 faced high out-of-pocket costs, which prevented initiation of therapy. This underscores the persistent impact of social determinants of health and inequities in access to guideline-directed care, even within structured intervention programs. In spite of the lack of an updated U.S. cost-effectiveness analysis, the available studies do highlight the favorable incremental cost-effectiveness ratio for PCSK9i, especially among high-risk patients.^42^ PCSK9i mAbs are also becoming a more feasible option as an initial nonstatin agent for patients with clinical ASCVD who are at very high risk or have multiple risk enhancers.^6^ Interestingly, analyses across other health systems—including those in Germany, the United Kingdom, Canada, Spain, China, Saudi Arabia, and Russia—have shown that PCSK9i mAbs (evolocumab and alirocumab) have a favorable economic profile, particularly for secondary prevention, and consistently demonstrate a more favorable cost–benefit ratio compared with other lipid-lowering agents such as icosapent ethyl, fibrates, and ezetimibe.^43^ Our findings highlight the effectiveness of an M2B program at improving LDL-C goal attainment in patients with established ASCVD postrevascularization. This approach enhances access to PCSK9i mAbs and may help to address barriers such as clinical inertia and suboptimal follow-up. The implementation of a bundled, proactive approach demonstrates the potential of structured programs to optimize secondary prevention of ASCVD. Future research should assess the long-term impact of M2B initiatives on CV outcomes and health care utilization across broader patient populations.

### Studt Limitations

Our study has several limitations. First, given the retrospective nature of the historical cohort and despite the matching efforts, there were variables not accounted for that could have impacted LDL-C testing and treatment in these patients. Second, since the cost of PCSK9i mAbs was drastically reduced in late 2018 and early 2019 and we included patients from January 2018, access to PCSK9i mAbs could have been theoretically lower for patients included in early 2018. Third, after direct matching, baseline LDL-C levels were significantly lower in the PCSK9i mAbs group. However, even after applying expanded matching to address these differences, the findings remained consistent. Fourth, there was a significant difference in follow-up LDL-C timing between the prospective and retrospective cohorts. In the M2B group, testing was protocolized and timely, whereas in the retrospective group, delayed testing reflected real-world practice, as previously shown by our group.^35^ Excluding patients with follow-up beyond 6 months would have introduced selection bias and limited generalizability. This difference should be considered when interpreting the results. Fifth, patients who did not initiate PCSK9i therapy due to insurance denial, high out-of-pocket costs, provider disagreement, or personal preference were not followed as part of the program and were excluded from outcome analyses, which may limit generalizability but also reflects persistent real-world challenges, even within structured interventions. Finally, we did not evaluate the impact of either strategy on major adverse cardiovascular events, as the study design, sample size, and follow-up duration were not originally powered to detect differences in clinical outcomes. In addition, we did not assess the changes in prescription rates before and after the intervention or examine their relationship with clinical outcomes. Nonetheless, LDL-C is a well-established surrogate endpoint, validated and accepted by the Food and Drug Administration as evidence of clinical benefit.^44^

## Conclusions

The implementation of an M2B program for early initiation of guideline-recommended PCSK9i mAbs in patients undergoing myocardial or peripheral artery revascularization was associated with enhanced testing and attainment of LDL-C and non–HDL-C goals in a real-world clinical practice cohort.

Perspectives **COMPETENCY IN PATIENT CARE AND PROCEDURAL SKILLS:** Implementation of a M2B program for early initiation of PCSK9 inhibitors in hospitalized patients with ASCVD undergoing revascularization supports timely adoption of guideline-recommended therapies. This structured, pharmacist-involved intervention improves medication access, adherence, and LDL-C goal attainment, reinforcing best practices in discharge planning and secondary prevention.

**TRANSLATIONAL OUTLOOK:** Despite strong guideline support, real-world uptake of PCSK9 inhibitors remains limited due to clinical inertia, system-level barriers, and insurance-related challenges. This study demonstrates that a structured M2B program can effectively address these gaps and improve lipid management in high-risk ASCVD patients. Future research should evaluate the long-term sustainability, cost-effectiveness, and broader implementation of such programs, particularly in underserved populations and diverse health care settings.

## Funding support and author disclosures

This study was sponsored by 10.13039/100002429Amgen. Drs Lorenzatti, Filtz, and Slipczuk are supported by institutional grants from 10.13039/100002429Amgen and 10.13039/100004320Philips. Dr Shapiro is supported by institutional grants from 10.13039/100002429Amgen, 10.13039/100014931Arrowhead, 10.13039/10002088789bio, 10.13039/100001003Boehringer Ingelheim, Cleerly, 10.13039/501100022336Esperion, Ionis, 10.13039/100004334Merck, New Amsterdam, and 10.13039/100008272Novartis and has participated in scientific advisory boards with Amgen, Arrowhead, Ionis, Merck, New Amsterdam, and Novartis and has served as a consultant for Aidoc, Arrowhead, Ionis, Kaneka, Novartis, Novo Nordisk, Regeneron, and Tourmaline. Dr Kalich, Jones, and Kathe are employees and stockholders in Amgen. Dr Gulati has served on advisory boards for Esperion, Medtronic, and Novartis. Dr Di Palo is supported by institutional grant from 10.13039/100004325AstraZeneca. All other authors have reported that they have no relationships relevant to the contents of this paper to disclose.

## References

[1] Vaduganathan M., Mensah G.A., Turco J.V., Fuster V., Roth G.A. (2022). The global burden of cardiovascular diseases and risk: a compass for future health. J Am Coll Cardiol.

[2] Silverman M.G., Ference B.A., Im K. (2016). Association between lowering LDL-C and cardiovascular risk reduction among different therapeutic interventions: a systematic review and meta-analysis. JAMA.

[3] Cholesterol Treatment Trialists’ (CTT) Collaboration, Baigent C., Blackwell L. (2010). Efficacy and safety of more intensive lowering of LDL cholesterol: a meta-analysis of data from 170,000 participants in 26 randomised trials. Lancet.

[4] Grundy S.M., Stone N.J., Bailey A.L. (2019). 2018 AHA/ACC/AACVPR/AAPA/ABC/ACPM/ADA/AGS/APhA/ASPC/NLA/PCNA guideline on the management of blood cholesterol: executive summary: a report of the American college of cardiology/american heart association task force on clinical practice guidelines. J Am Coll Cardiol.

[5] Mach F., Baigent C., Catapano A.L. (2020). 2019 ESC/EAS guidelines for the management of dyslipidaemias: lipid modification to reduce cardiovascular risk. Eur Heart J.

[6] Writing Committee, Lloyd-Jones D.M., Morris P.B. (2022). 2022 ACC expert consensus decision pathway on the role of nonstatin therapies for LDL-cholesterol lowering in the management of atherosclerotic cardiovascular disease risk: a report of the American college of cardiology solution set oversight committee. J Am Coll Cardiol.

[7] Sabatine M.S., Giugliano R.P., Keech A.C. (2017). Evolocumab and clinical outcomes in patients with cardiovascular disease. N Engl J Med.

[8] Cannon C.P., Blazing M.A., Giugliano R.P. (2015). Ezetimibe added to statin therapy after acute coronary syndromes. N Engl J Med.

[9] Nissen S.E., Lincoff A.M., Brennan D. (2023). Bempedoic acid and cardiovascular outcomes in statin-intolerant patients. N Engl J Med.

[10] LaRosa J.C., Grundy S.M., Waters D.D. (2005). Intensive lipid lowering with atorvastatin in patients with stable coronary disease. N Engl J Med.

[11] Lowenstern A.M., Li S., Navar A.M. (2018). Measurement of low-density lipoprotein cholesterol levels in primary and secondary prevention patients: insights from the PALM registry. J Am Heart Assoc.

[12] Wong N.D., Young D., Zhao Y. (2016). Prevalence of the American college of cardiology/american heart association statin eligibility groups, statin use, and low-density lipoprotein cholesterol control in US adults using the national health and nutrition examination survey 2011-2012. J Clin Lipidol.

[13] Cannon C.P., De Lemos J.A., Rosenson R.S. (2021). Use of lipid-lowering therapies over 2 years in GOULD, a registry of patients with atherosclerotic cardiovascular disease in the US. JAMA Cardiol.

[14] Underberg J., Toth P.P., Rodriguez F. (2022). LDL-C target attainment in secondary prevention of ASCVD in the United States: barriers, consequences of nonachievement, and strategies to reach goals. Postgrad Med.

[15] Stedge B., Xu J., Kubes J.N. (2023). Meds to beds at hospital discharge improves medication adherence and readmission rates in select populations. South Med J.

[16] Giugliano R.P., Desai N.R., Kohli P. (2012). Efficacy, safety, and tolerability of a monoclonal antibody to proprotein convertase subtilisin/kexin type 9 in combination with a statin in patients with hypercholesterolaemia (LAPLACE-TIMI 57): a randomised, placebo-controlled, dose-ranging, phase 2 study. Lancet.

[17] Raal F.J., Stein E.A., Dufour R. (2015). PCSK9 inhibition with evolocumab (AMG 145) in heterozygous familial hypercholesterolaemia (RUTHERFORD-2): a randomised, double-blind, placebo-controlled trial. Lancet.

[18] Schwartz G.G., Steg P.G., Szarek M. (2018). Alirocumab and cardiovascular outcomes after acute coronary syndrome. N Engl J Med.

[19] Jia X., Al Rifai M., Saeed A., Ballantyne C.M., Virani S.S. (2022). PCSK9 inhibitors in the management of cardiovascular risk: a practical guidance. Vasc Health Risk Manag.

[20] Karalis D.G., Mallya U.G., Ghannam A.F., Elassal J., Gupta R., Boklage S.H. (2018). Prescribing patterns of proprotein convertase subtilisin-kexin type 9 inhibitors in eligible patients with clinical atherosclerotic cardiovascular disease or heterozygous familial hypercholesterolemia. Am J Cardiol.

[21] Myers K.D., Farboodi N., Mwamburi M. (2019). Effect of access to prescribed PCSK9 inhibitors on cardiovascular outcomes. Circ Cardiovasc Qual Outcomes.

[22] Zafrir B., Egbaria A., Stein N., Elis A., Saliba W. (2021). PCSK9 inhibition in clinical practice: treatment patterns and attainment of lipid goals in a large health maintenance organization. J Clin Lipidol.

[23] Ohdsi – observational health data sciences and informatics. https://www.ohdsi.org/.

[24] OMOP common data model. https://ohdsi.github.io/CommonDataModel/.

[25] Ward R., Hallinan C.M., Ormiston-Smith D., Chidgey C., Boyle D. (2024). The OMOP common data model in Australian primary care data: building a quality research ready harmonised dataset. PLoS One.

[26] Southern W.N., Bellin E.Y., Arnsten J.H. (2011). Longer lengths of stay and higher risk of mortality among inpatients of physicians with more years in practice. Am J Med.

[27] Ouchi Y., Sasaki J., Arai H. (2019). Ezetimibe lipid-lowering trial on prevention of atherosclerotic cardiovascular disease in 75 or older (EWTOPIA 75): a randomized, controlled trial. Circulation.

[28] Rosenson R.S., Farkouh M.E., Mefford M. (2017). Trends in use of high-intensity statin therapy after myocardial infarction, 2011 to 2014. J Am Coll Cardiol.

[29] Bradley E.H., Curry L., Horwitz L.I. (2013). Hospital strategies associated with 30-day readmission rates for patients with heart failure. Circ Cardiovasc Qual Outcomes.

[30] Agarwal P., Poeran J., Meyer J., Rogers L., Reich D.L., Mazumdar M. (2019). Bedside medication delivery programs: suggestions for systematic evaluation and reporting. Int J Qual Health Care.

[31] El Hajj M.S., Jaam M.J., Awaisu A. (2018). Effect of pharmacist care on medication adherence and cardiovascular outcomes among patients post-acute coronary syndrome: a systematic review. Res Social Adm Pharm.

[32] Marco-Benedí V., Sánchez-Hernández R.M., Díaz J.L. (2024). PCSK9 inhibitors on the management of primary and secondary cardiovascular prevention. Lipids Health Dis.

[33] Kim O.M., Givens T.K., Tang E.G. (2023). Real-world outcomes of proprotein convertase subtilisin Kexin-9 inhibitor use. J Cardiovasc Pharmacol.

[34] Colantonio L.D., Wang Z., Jones J. (2024). Low-density lipoprotein cholesterol testing following myocardial infarction hospitalization among medicare beneficiaries. JACC Adv.

[35] Greenberg G., Lorenzatti D., Filtz A. (2025). Disparities in achieving LDL-C goals after revascularization in a diverse real-world cohort. Eur J Prev Cardiol.

[36] MacDougall D.E., Baum S.J., Ahmed C.D., McGowan M.P., Wilemon K.A. (2024). Trends in patient access to and utilization of prescribed PCSK9 inhibitors in a large US claims database from 2015 to 2021. Circ Cardiovasc Qual Outcomes.

[37] Zhang X.-L., Zhu Q.-Q., Zhu L. (2015). Safety and efficacy of anti-PCSK9 antibodies: a meta-analysis of 25 randomized, controlled trials. BMC Med.

[38] Brandts J., Bray S., Villa G. (2023). Optimal implementation of the 2019 ESC/EAS dyslipidaemia guidelines in patients with and without atherosclerotic cardiovascular disease across Europe: a simulation based on the DA VINCI study. Lancet Reg Health Eur.

[39] Pencina K.M., Thanassoulis G., Wilkins J.T. (2019). Trajectories of Non-HDL cholesterol across midlife: implications for cardiovascular prevention. J Am Coll Cardiol.

[40] Visseren F.L.J., Mach F., Smulders Y.M. (2021). 2021 ESC guidelines on cardiovascular disease prevention in clinical practice. Eur Heart J.

[41] Schubert J., Leosdottir M., Lindahl B. (2024). Intensive early and sustained lowering of non–high-density lipoprotein cholesterol after myocardial infarction and prognosis: the SWEDEHEART registry. Eu Heart J.

[42] Fonarow G.C., van Hout B., Villa G., Arellano J., Lindgren P. (2019). Updated cost-effectiveness analysis of evolocumab in patients with very high-risk atherosclerotic cardiovascular disease. JAMA Cardiol.

[43] Mercep I., Strikic D., Hrabac P., Pecin I., Reiner Ž. (2024). PCSK9 inhibition: from effectiveness to cost-effectiveness. Front Cardiovasc Med.

[44] FDA facts: biomarkers and surrogate endpoints | FDA. https://www.fda.gov/about-fda/innovation-fda/fda-facts-biomarkers-and-surrogate-endpoints.

