# Supplementary material for: Impact of a Meds-to-Beds PCSK9i Initiation Program on LDL-C in Patients Undergoing ASCVD Revascularization
Source: JACC Adv. 2025 Aug 19;4(9):102075. doi: 10.1016/j.jacadv.2025.102075 (PMC12395049; doi:10.1016/j.jacadv.2025.102075)
Supplement: Supplementary Material [file mmc1.docx]

Supplemental Table 1. SPIRIT 2013 Checklist – ELL-ASCVD Study

| SPIRIT Item | Description | Location in Manuscript |
| --- | --- | --- |
| 1. Title | Descriptive title identifying the study design, population, and interventions | Title Page |
| 2a. Trial registration | Not applicable (real-world implementation trial, not registered on ClinicalTrials.gov) | Response to Reviewer |
| 2b. Trial identifier | Not applicable | – |
| 3. Protocol version | Not applicable (not a registered protocol trial) | – |
| 4. Funding | Funding source and role (Amgen) | Title Page / Funding Section |
| 5. Roles and responsibilities | Authors and institutional affiliations provided | Title Page / Author List |
| 6a. Background & rationale | Scientific background and rationale for the intervention | Introduction |
| 6b. Explanation for comparators | Retrospective matched cohort used for comparison | Methods – Study Design / Retrospective Cohort |
| 7. Objectives | Study objective to evaluate LDL-C goal attainment post-PCSK9i via M2B program | Abstract / Introduction |
| 8. Trial design | Prospective, open-label, interventional, single-center study | Methods – Study Design |
| 9. Study setting | Single large quaternary academic health system (Montefiore-Einstein) | Methods – Study Setting |
| 10. Eligibility criteria | Inclusion/exclusion criteria detailed | Methods – Prospective Cohort |
| 11a. Interventions | Description of PCSK9i meds-to-beds implementation strategy | Methods – M2B Program |
| 12. Outcomes | Primary and secondary LDL-C and non-HDL-C outcomes defined | Methods – Outcomes |
| 13. Participant timeline | 6-month follow-up window post-revascularization | Methods – Follow-Up |
| 14. Sample size | Not based on formal power calculation | Not explicitly stated |
| 15. Recruitment | Convenience sample from consecutive admissions (June 2023–Feb 2024) | Methods – Prospective Cohort |
| 16a. Allocation | Not randomized; prospective vs. retrospective matched cohort | Methods – Matching Algorithms |
| 20a. Statistical methods | Statistical plan described: Wilcoxon, Chi-squared, logistic regression | Methods – Statistical Analysis |
| 21. Data monitoring | Not applicable (minimal risk QI study, no DSMB) | – |
| 22. Harms | Not assessed; real-world use of FDA-approved therapy | Limitations (implicitly) |
| 23. Auditing | Not applicable | – |
| 24. Research ethics approval | IRB approval obtained (Albert Einstein College of Medicine) | Methods – Study Design |
| 25. Protocol amendments | Not applicable | – |
| 26a. Consent or assent | Written/verbal informed consent for all prospective participants | Methods – Study Design |
| 27. Confidentiality | Not explicitly discussed | – |
| 28. Declaration of interests | Disclosures provided | Title Page / Disclosures Section |
| 29. Data access | Data available upon reasonable request | Data Sharing Statement |
| 30. Dissemination policy | Results disseminated via peer-reviewed publication | Manuscript Submission |

Supplemental Table 2. Baseline characteristics and outcomes using an alternative matching model^a^

|  | **PCSK9i mAbs Group**  N=73^b^ | **Control Group**  N=136^b^ | **Overall**  N=209^b^ | **p-value**^c^ |
| --- | --- | --- | --- | --- |
| **Age (years)** | 66 (61, 71) | 66 (60, 71) | 66 (60, 71) | >0.9 |
| **Sex** |  |  |  | 0.4 |
| Female | 26 (36%) | 41 (30%) | 67 (32%) |  |
| Male | 47 (64%) | 95 (70%) | 142 (68%) |  |
| **Procedure** |  |  |  | 0.3 |
| PCI | 50 (68%) | 100 (74%) | 150 (72%) |  |
| CABG | 13 (18%) | 26 (19%) | 39 (19%) |  |
| PAD | 10 (14%) | 10 (7.4%) | 20 (9.6%) |  |
| **Race/Ethnicity** |  |  |  | 0.5 |
| Hispanic | 42 (58%) | 75 (55%) | 117 (56%) |  |
| Non-Hispanic Black | 11 (15%) | 30 (22%) | 41 (20%) |  |
| Non-Hispanic White | 10 (14%) | 12 (8.8%) | 22 (11%) |  |
| Other/Unknown | 10 (14%) | 19 (14%) | 29 (14%) |  |
| **Insurance Type** |  |  |  | 0.2 |
| Medicare | 25 (34%) | 44 (32%) | 69 (33%) |  |
| Medicaid | 25 (34%) | 51 (38%) | 76 (36%) |  |
| Private | 15 (21%) | 36 (26%) | 51 (24%) |  |
| Unknown | 8 (11%) | 5 (3.7%) | 13 (6.2%) |  |
| **Hypertension** | 68 (93%) | 135 (99%) | 203 (97%) | **0.021** |
| **Diabetes Mellitus** | 52 (71%) | 106 (78%) | 158 (76%) | 0.3 |
| **Obesity** | 32 (44%) | 55 (40%) | 87 (42%) | 0.6 |
| **CAD** | 70 (96%) | 136 (100%) | 206 (99%) | **0.041** |
| **Myocardial Infarction** | 38 (52%) | 113 (83%) | 151 (72%) | **<0.001** |
| **CHF** | 32 (44%) | 85 (63%) | 117 (56%) | **0.010** |
| **Atrial Fibrillation** | 13 (18%) | 31 (23%) | 44 (21%) | 0.4 |
| **Peripheral Vascular Disease** | 28 (38%) | 58 (43%) | 86 (41%) | 0.5 |
| **Chronic Kidney Disease** | 32 (44%) | 56 (41%) | 88 (42%) | 0.7 |
| **Chronic Lung Disease** | 32 (44%) | 61 (45%) | 93 (44%) | 0.9 |
| **Substance Use** | 23 (32%) | 58 (43%) | 81 (39%) | 0.12 |
| **Baseline Statin** | 66 (90%) | 136 (100%) | 202 (97%) | **<0.001** |
| **Baseline High-intensity Statin** | 65 (89%) | 136 (100%) | 201 (96%) | **<0.001** |
| **Baseline Ezetimibe** | 0 (NA%) | 19 (14%) | 19 (14%) | >0.9 |
| **Follow-up Statin** | 63 (86%) | 132 (97%) | 195 (93%) | **0.006** |
| **Follow-up High-intensity Statin** | 62 (85%) | 127 (93%) | 189 (90%) | **0.048** |
| **Follow-up Ezetimibe** | 0 (NA%) | 27 (20%) | 27 (20%) | >0.9 |
| **Follow-up PCSK9i** | 0 (NA%) | 8 (5.9%) | 8 (5.9%) | >0.9 |
| **Baseline LDL-C** | 95 (80, 121) | 97 (78, 130) | 96 (79, 129) | >0.9 |
| **Baseline Non–HDL-C** | 117 (102, 160) | 122 (104, 160) | 119 (103, 160) | 0.7 |
| **Baseline HDL-C** | 40 (34, 47) | 41 (32, 48) | 40 (34, 48) | 0.8 |
| **Baseline Total Cholesterol** | 160 (140, 200) | 162 (143, 201) | 161 (143, 201) | 0.8 |
| **Baseline Triglycerides** | 107 (79, 144) | 120 (90, 161) | 112 (85, 153) | 0.064 |
| **Follow-up LDL-C** | 33 (23, 50) | 77 (64, 104) | 67 (40, 90) | **<0.001** |
| **Follow-up Non–HDL-C** | 59 (45, 89) | 102 (83, 133) | 93 (68, 126) | **<0.001** |
| **Follow-up HDL-C** | 41 (37, 49) | 39 (34, 46) | 41 (35, 47) | 0.10 |
| **Follow-up Total Cholesterol** | 105 (86, 129) | 141 (124, 177) | 131 (105, 171) | **<0.001** |
| **Follow-up Triglycerides** | 112 (88, 151) | 108 (82, 147) | 108 (83, 148) | 0.2 |
| **Follow-up LDL-C Within 90 Days** | 43 (59%) | 34 (25%) | 77 (37%) | **<0.001** |
| **Follow-up LDL-C Within 6 Months** | 65 (89%) | 51 (38%) | 116 (56%) | **<0.001** |
| **Follow-up LDL-C Within 1 Year** | 72 (99%) | 71 (52%) | 143 (68%) | **<0.001** |
| **Months to Follow-up LDL-C** | 3 (2, 4) | 12 (4, 23) | 5 (2, 16) | **<0.001** |
| **Follow-up LDL-C <70 mg/dL** | 66 (90%) | 47 (35%) | 113 (54%) | **<0.001** |
| **Follow-up LDL-C <55 mg/dL** | 57 (78%) | 23 (17%) | 80 (38%) | **<0.001** |
| **Change in LDL-C (mg/dL)** | −59 (−88, −43) | −16 (−56, 5) | −37 (−68, 0) | **<0.001** |
| **Reduction in LDL-C (%)** | 66 (50, 78) | 17 (−6, 46) | 37 (0, 64) | **<0.001** |
| **Follow-up Non–HDL-C <100 mg/dL** | 49 (80%) | 61 (45%) | 110 (56%) | **<0.001** |
| **Follow-up Non–HDL-C <85 mg/dL** | 45 (74%) | 39 (29%) | 84 (43%) | **<0.001** |

^a^Match Algorithm: Exact – Procedure Type. Nearest (Propensity Score) – Age, Age^2^, Gender, Ethnicity/Race, Insurance Type, HTN, DM, Obesity, CAD, MI, CHF, Afib, PVD, CKD, Lung Disease, Substance Use, Baseline LDL-C, (baseline LDL-C)^^2^ Ratio: 2 to 1; ^b^Median (Q1, Q3) or n (%); ^c^Wilcoxon rank-sum test, Pearson's Chi-squared test, or Fisher's exact test.

Afib: atrial fibrillation; CABG: coronary artery bypass graft; CAD: coronary artery disease; CHF: congestive heart failure; CKD: chronic kidney disease; DM: diabetes mellitus; HDL-C: high-density lipoprotein cholesterol; HTN: hypertension; LDL-C: low-density lipoprotein cholesterol; mAbs: monoclonal antibodies; MI: myocardial infarction; PAD: peripheral artery disease; PCI: percutaneous coronary intervention; PCSK9i: proprotein convertase subtilisin/kexin type 9 inhibitor; PVD: peripheral vascular disease.

Supplemental Table 3. Adjusted multivariable logistic regression assessing the impact of the intervention on LDL-C goal attainment, excluding baseline cholesterol and non–HDL-C due to collinearity with LDL-C.

| **Characteristic** | **OR***^1^* | **95% CI***^1^* | **p-value** |
| --- | --- | --- | --- |
| Treatment Group | 65.2 | 15.9, 402 | **<0.001** |
| Age (years) | 1.05 | 1.00, 1.12 | 0.068 |
| Sex |  |  |  |
| Female | — | — |  |
| Male | 1.50 | 0.62, 3.68 | 0.4 |
| Procedure |  |  |  |
| PCI | — | — |  |
| CABG | 0.32 | 0.10, 0.91 | **0.038** |
| PAD | 0.33 | 0.07, 1.57 | 0.2 |
| Race/Ethnicity |  |  |  |
| Hispanic | — | — |  |
| Non-Hispanic Black | 0.78 | 0.30, 1.97 | 0.6 |
| Non-Hispanic White | 0.99 | 0.25, 4.02 | >0.9 |
| Other/Unknown | 1.81 | 0.58, 5.80 | 0.3 |
| Insurance Type |  |  |  |
| Medicare | — | — |  |
| Medicaid | 0.60 | 0.18, 1.99 | 0.4 |
| Private | 0.61 | 0.17, 2.15 | 0.4 |
| Unknown | 0.53 | 0.09, 3.74 | 0.5 |
| Hypertension | 6.45 | 0.44, 93.8 | 0.2 |
| Diabetes Mellitus | 1.03 | 0.39, 2.74 | >0.9 |
| Obesity | 1.02 | 0.45, 2.35 | >0.9 |
| CAD | 16.6 | 0.34, 844 | 0.13 |
| Myocardial Infarction | 0.61 | 0.23, 1.56 | 0.3 |
| CHF | 0.68 | 0.28, 1.59 | 0.4 |
| Atrial Fibrillation | 1.81 | 0.64, 5.26 | 0.3 |
| Peripheral Vascular Disease | 1.10 | 0.48, 2.51 | 0.8 |
| Chronic Kidney Disease | 0.73 | 0.32, 1.66 | 0.4 |
| Chronic Lung Disease | 0.90 | 0.37, 2.18 | 0.8 |
| Substance Use | 1.48 | 0.63, 3.52 | 0.4 |
| Baseline High Intensity Statin | 0.00 | N/A | >0.9 |
| Baseline Ezetimibe | 0.25 | 0.06, 0.88 | **0.042** |
| Baseline LDL-C | 1.00 | 0.99, 1.01 | 0.9 |
| Baseline Triglycerides | 1.00 | 1.00, 1.01 | 0.5 |
| *^1^*OR = Odds Ratio, CI = Confidence Interval | | | |

**Supplemental Table 4.** Multivariable Poisson regression estimating the effect of the intervention on LDL-C goal attainment.

| **Characteristic** | **RR***^1^* | **95% CI***^1^* | **p-value** |
| --- | --- | --- | --- |
| Treatment Group | 2.39 | 1.59, 3.62 | **<0.001** |
| Hypertension | 1.24 | 0.50, 4.12 | 0.7 |
| Myocardial Infarction | 0.90 | 0.61, 1.35 | 0.6 |
| Baseline High Intensity Statin | 0.97 | 0.49, 2.24 | >0.9 |
| Baseline Ezetimibe | 0.72 | 0.41, 1.20 | 0.2 |
| Baseline LDL-C | 1.00 | 0.99, 1.01 | >0.9 |
| Baseline Triglycerides | 1.00 | 1.00, 1.00 | 0.7 |
| *^1^*RR = Risk Ratio, CI = Confidence Interval | | | |

Supplemental Figure 1. Covariate balance before and after matching using the primary (A) and alterative (B) matching models.


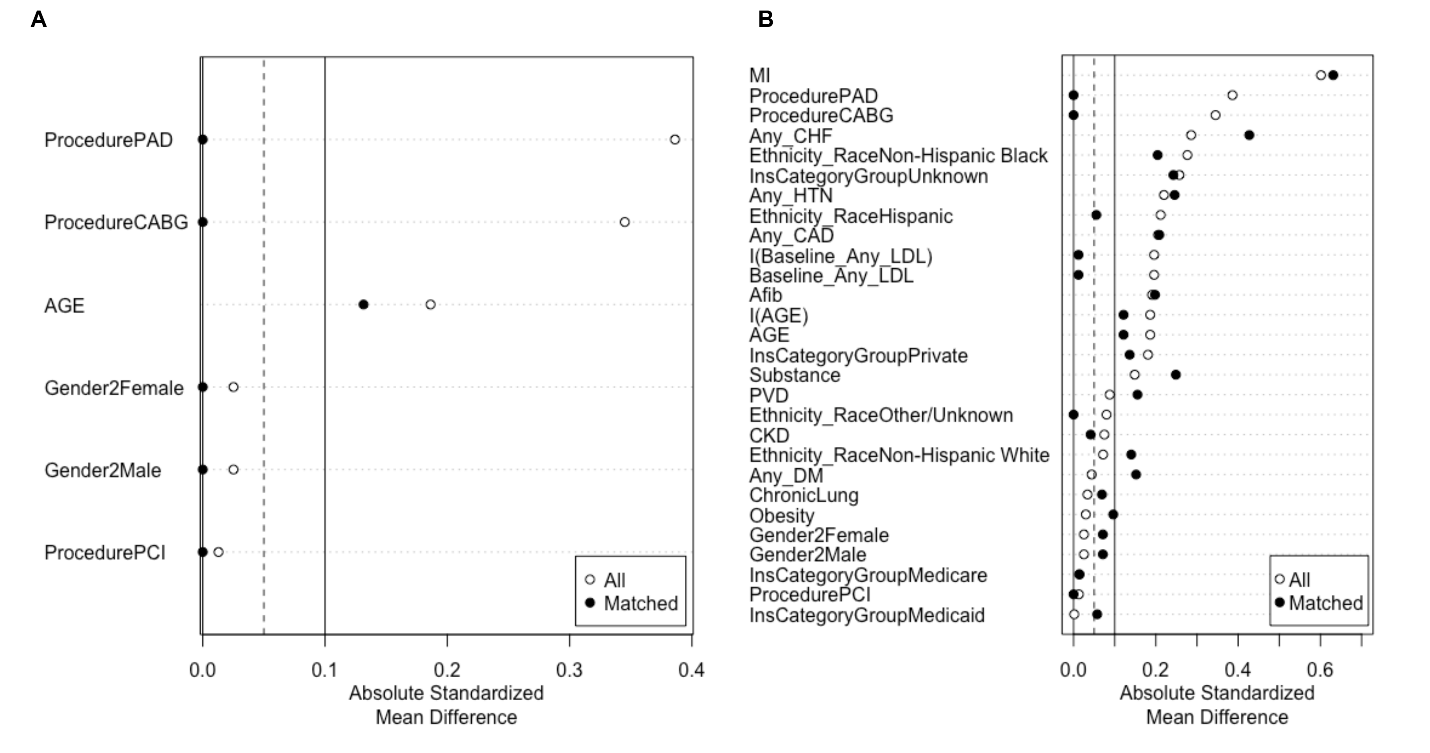


Love plot displaying absolute standardized mean differences for baseline variables before and after matching (1:2 ratio)
